# First Report of Protective Activity of *Paronychia argentea* Extract against Tobacco Mosaic Virus Infection

**DOI:** 10.3390/plants10112435

**Published:** 2021-11-11

**Authors:** Ahmed Abdelkhalek, Abdulaziz A. Al-Askar, Maha M. Alsubaie, Said I. Behiry

**Affiliations:** 1 Plant Protection and Biomolecular Diagnosis Department, ALCRI, City of Scientific Research and Technological Applications, New Borg El Arab City, Alexandria 21934, Egypt; 2 Botany and Microbiology Department, Faculty of Science, King Saud University, Riyadh 11451, Saudi Arabia; 441203885@student.ksu.ed.sa; 3 Agricultural Botany Department, Faculty of Agriculture (Saba Basha), Alexandria University, Alexandria 21531, Egypt; said.behiry@alexu.edu.eg

**Keywords:** *Paronychia argentea*, plant extract, TMV, tomato, antioxidant enzymes, gene expression, HPLC

## Abstract

The widespread use of chemical control agents and pesticides for plant-pathogen control has caused many human health and environmental issues. Plant extracts and biocontrol agents have robust antimicrobial activity against different plant pathogens. However, their antiviral activities are still being investigated. In the present study, the methanol extract of *Paronychia argentea* was characterized and evaluated for its protective activity against the tobacco mosaic virus (TMV) infection in tomato plants under greenhouse conditions at 21 days post-inoculation. The results showed that the foliar application of *P. argentea* extract (10 µg/mL) enhanced tomato plant growth, resulting in significant increases in shoot and root parameters and total chlorophyll contents. Moreover, a significant reduction in TMV accumulation level in *P. argentea*-treated plants of 77.88% compared to non-treated plants was reported. Furthermore, induction of systemic resistance with significant elevation in production of antioxidant enzymes (PPO, CAT, and SOD) and transcriptional levels of the pathogenesis-related proteins (PR-1 and PR-7) and polyphenolic genes (CHS and HQT) were also observed. Out of 16 detected compounds, HPLC analysis revealed that the most abundant polyphenolic compounds found in *P. argentea* extract were gallic acid (5.36 µg/mL), kaempferol (7.39 µg/mL), quercetin (7.44 µg/mL), ellagic acid (7.89 µg/mL), myricetin (8.36 µg/mL), and ferulic acid (8.69 µg/mL). The findings suggest that the use of *P. argentea* extract as an effective and safe source for the production of bioactive compounds may offer a solution for a promising approach for the management of plant viral infections. To the best of our knowledge, this is the first report of the protective activity of *P. argentea* extract against plant viral diseases.

## 1. Introduction 

Globally, plant viral diseases are a significant threat to food crop production. Among them, the tobacco mosaic virus (TMV, genus *Tobamovirus*), a single-strand of RNA virus, infects 66 families (approximately 900 species), especially plants in the Solanaceae family such as tobacco and tomato [1,2]. After the potato, the tomato (*Lycopersicon esculentum* L.) is the world’s second most consumed vegetable crop [3]. Generally, TMV induces a systemic infection and morphological changes associated with mosaic and/or chlorotic lesions on infected plants’ leaves, resulting in a complete loss of crop productivity [4,5]. In addition, by preventing the growth of reproductive organs, particularly flowering organs, this disease can cause up to 100% crop loss [6]. TMV is difficult to control because it is easily and rapidly spread where it is mechanically transferred, and symptoms appear 7 to 12 days after infection in susceptible plants [7].

*Paronychia argentea* L. is a perennial plant that belongs to the family of Caryophyllaceae in habitats of the Mediterranean area. The literature review showed the great medicinal bioactivities of *P. argentea*. Its extract has antioxidant, antimicrobial, and nephroprotective activities [8]. The application of plant extracts, secondary metabolites as biocontrol agents for reducing and overcoming plant viral infections, has become an effective alternative control method since it is safe and environmentally friendly and restricts chemical control uses [9]. Aqueous and ethanolic extracts of *P. argentea* aerial parts show antimicrobial efficacy against the majority of *Candida* strains and Gram-positive bacteria, such as *Helicobacter pylori* [10,11]. Several plant extracts including *Boerhaavia diffusa*, *Boerhaavia diffusa*, *Clerodendrum aculeatum*, *Eucaluptus camaldulensis*, *Haplophyllum tuberculatum, Potentilla arguta*, *Mirabilis jalapa*, *Sambucus racemose*, and *Thuja orientalis* exhibited an inhibitory effect against infection by plant viruses [12,13]. High-performance liquid chromatography (HPLC)-UV/DAD and HPLC-ESI-MSn analysis of *P. argentea* extract defined different bioactive phytochemical compounds such as flavonoids, vanillic acid, luteolin, and quercetin [14]. The potential of plant extracts to induce systemic resistance and inhibit viral replication could be due to their high content of flavonoids and phenolic acids, and it might explain their antioxidant activity and antimicrobial efficacy [8]. Additionally, it was reported that the application of plant extracts was associated with the induction of pathogenesis-related proteins, phenylalanine ammonia-lyase (PAL), peroxidase (POD), and polyphenol oxidase (PPO) upon viral infection [15]. However, this field is still insufficiently explored, necessitating more data to comprehensively understand the different mechanisms by which these plant extracts act as antiviral agents.

The current study aimed to examine the protective efficacy of *Paronychia argentea* methanolic extract against TMV for the first time. The transcriptional level changes of three tomato pathogenesis-related proteins (PR-1, PR-2, and PR-7) and two polyphenolic genes, chalcone synthase (CHS) and hydroxycinnamoyl Co A quinate hydroxycinnamoyl transferase (HQT), as well as the accumulation level of TMV inside infected tissues, were evaluated. Moreover, the main phytochemical constituents of *P. argentea* were analyzed by using HPLC.

## 2. Materials and Methods

### 2.1. Preparation of the Paronychia argentea Extract

Ten *Paronychia argentea* plants were collected during the summer of 2020 from Bir El Abd, North Sinai Governorate, Egypt, at district 31°02’06.8” N, 33°00’03.2” E. The plants’ aerial parts were washed with tap water, cleaned and air-dried at the laboratory room temperature of 25 °C for one week, then ground to powder using a laboratory mill (FOSS Mill, CT 293 Cyclotec, Hilleroed, Denmark) and mixed. A 100 g powder mix was soaked in 200 mL of 96% methanol alcohol and shaken at 200 rpm (Orbital shaker VWR, Radnor, Pennsylvania, USA) in a dark bottle for a week. The methanol extract was filtered through Whatman no.1 filter paper. The filtrate was concentrated using a rotary evaporator (RE-301 3L, Henan Lanphan Industry Co., Ltd., Zhengzhou, Henan, China). 

### 2.2. HPLC Analysis of P. argentea Extract Polyphenolic Compounds

HPLC, conducted with a Zorbax Eclipse Plus C18 (RP18, ODS, Octadecyl) column (95 Å, 3.5 µm, 4.6 × 100 mm), was used to determine the phenolic and flavonoids content in *P. argentea* extract [16]. The analysis was carried out based on the 16 standard phenolic and flavonoid compounds: syringenic acid, *p*-coumaric acid, caffeic acid, pyrogallol acid, ferulic acid, gallic acid, ellagic acid, rutin, naringin, quercetin, kaempferol, hesperidin, catechin, 7-OH flavone, apigenin, and myricetin (Sigma-Aldrich, St. Louis, MO, USA).

### 2.3. Source of TMV Isolate

The tobacco mosaic virus (TMV) strain KH1 (accession number MG264131) was isolated before from TMV-infected tomato plants and maintained on tobacco plants [17]. The purified TMV inoculum was diluted to 20 μg/mL in 100 mM sodium phosphate buffer, pH 7 before use.

### 2.4. Greenhouse Experiment and Antiviral Protective Activity Assay

The antiviral activity of *P. argentea* extract (10 μg/mL) was tested first on *Chenopodium amaranticolor* plants, which served as a TMV-local lesion host. The percentage of inhibition was estimated based on the number of local lesions generated on the leaves [13]. After the tomato-free virus seeds (Peto 86 cultivar) were surface sterilized, they were grown under insect-proof greenhouse conditions in plastic pots (25 cm) filled with sterilized clay and sand soil (1:1). The tomato seedlings were transferred into new pots after 28 days of seed growth. The TMV-mechanical inoculation of each tomato seedling was done after one week, as described earlier [5,18]. The experiment was composed of four different treatments, each of which had three repetitions and three tomato plants in each pot. The first treatment consisted of mock-tomato plants (control (T1)) treated with the inoculation buffer and sprayed with sterile double distilled water. The second treatment contained tomato plants mechanically inoculated with TMV (infected (T2)). The third treatment included tomato plants, foliar sprayed with 10 μg/mL of *P. argentea* extract (T3). The fourth treatment consisted of tomato plants that were foliar sprayed with 10 μg/ mL of *P. argentea* extract 24 h before mechanical inoculation of TMV (T4). All plants were housed in an insect-proof greenhouse at a temperature of 28 °C/16 °C (day/night) and relative humidity of 70%. The plants were monitored daily to observe symptom development. At 21 days post-inoculation (dpi), three biological replicates of tomato leaves from three different plants were harvested; then, enzyme activities and RNA extraction were determined for all treatments. For each treatment, the fresh and dry weights of the shoots and root systems were measured (g), and total chlorophyll content was determined (SPAD).

### 2.5. Enzyme Activity Determination

#### 2.5.1. Oxidative Stress Markers

##### Malondialdehyde Assay (MDA)

Malondialdehyde content (MDA) was determined by thiobarbituric acid (TBA) [19] in all tomato treatments. Tomato leaves were grounded in trichloroacetic acid (TCA, 0.1%) and centrifuged at 12,000 g for 30 min. After that, in a water bath (95 °C, 30 min), 1 mL of the resulting supernatant was mixed with the solution containing trichloroacetic acid (TCA) (20%): TBA (5%). The mixture was immediately cooled on ice. The product absorbance was measured at 600 nm, and the MDA concentration was expressed as µmol/g FW.

##### Hydrogen Peroxide Assay (H_2_O_2_)

H_2_O_2_ content in tomato leaves was measured according to Velikova et al. [20]. Briefly, small portions of tomato leaves were homogenized with 0.1% TCA. After centrifugation at 12,000 g for 15 min, equal volumes of the supernatant, KH_2_PO_4_ (10 mM, pH 7), and 1 M KI (1:1:1, *v*/*v*/*v*) were mixed and incubated at room temperature for 20 min. After that, the absorbance was measured at 390 nm. The amount of H_2_O_2_ was represented as µmol/g FW.

#### 2.5.2. Antioxidant Enzymes

For all enzyme activities reactions, leaf samples were homogenized with phosphate buffer pH 7 and centrifuged at 10,000 g, and the supernatant was used for further studies.

##### Polyphenol Oxidase (PPO)

PPO activity was measured according to the method of Cho and Ahn [21]. An amount of 500 µL of enzyme extract was incubated for 10 min at room temperature with a mixture of 1 mL of Tris-HCl and quinone (50 mM, pH 6). All measurements were carried out three times at 420 nm and represented as µmol/g FW.

##### Catalase (CAT) Activity

The method of Cakmak and Marschner [22] with minor modifications was used to determine CAT activity by measuring the rate of disappearance of H_2_O_2_ at 240 nm. A 50 µL enzyme extract was incubated with 1 mL of a mixture of potassium phosphate buffer (25 mM, pH 7) and H_2_O_2_ (10 mM as final concentration). The activity of CAT was measured as µmol/g FW.

##### Superoxide Dismutase (SOD) Activity

The ability of SOD to prevent the photochemical reduction of nitro blue tetrazolium (NBT) was measured using the procedure of Beauchamp and Fridovich [23] with slight changes. The reaction mixture contained KH_2_PO_4_ (50 mM, pH 7.8), EDTA (0.1 mM), L-methionine (10 mM), NBT (75 mM), and riboflavin (20 mM) reacted with 100 µL of enzyme extract in the tube. The tubes were incubated at 25 °C for 15 min under two 15 W fluorescent lamps before being placed in the dark. After that, the absorbance at 560 nm was measured. SOD activity was expressed as µmol/g FW.

### 2.6. Analysis of the Transcriptional Levels of the Defense-Related Genes Using Quantitative Real-Time PCR (qRT-PCR)

#### 2.6.1. RNA Extraction and cDNA Synthesis

Each treatment’s leaves of three biological replicates were collected at 21 dpi and kept at −80 °C until use. According to the manufacturer’s instructions, the total RNA was extracted by an RNeasy Plant Mini Kit (QIAGEN, Hilden, Germany). Each biological sample was a mix of nine samples derived from nine different plants. The extracted RNA was dissolved in DEPC-treated water, treated with RNase-free DNase to eliminate genomic DNA, had its concentration and purity quantified using Nano equipment (SPECTROstar, Ortenberg, Germany), and had its integrity assessed by agarose gel electrophoresis. An amount of 1 µg of RNA for each sample was used as a template and was reverse transcribed to cDNA using oligo (dT) and random hexamer primers with reverse transcriptase enzyme Super-Script II (Invitrogen, Waltham, MA, USA), according to the manufacturer’s instructions [24]. The reverse transcriptase reaction was performed at 42 °C for 1 h and deactivated at 80 °C for 5 min in a thermal cycler (Eppendorf, Hamburg, Germany). After holding at 4 °C, the PCR product was kept at −20 °C until used as a qRT-PCR template.

#### 2.6.2. qRT-PCR Assays

The accumulation levels of the TMV-CP gene and the transcriptional levels of three tomato pathogenesis-related proteins (PR-1, PR-2, and PR-7), as well as two polyphenolic genes (CHS and HQT), were quantified and analyzed using the qRT-PCR technique (Table 1). To normalize the expression levels of the different genes, the *β*-actin gene (Table 1) was employed as a housekeeping reference gene [25]. Each biological treatment was done on real-time equipment (Rotor-Gene 6000, QIAGEN, Germantown, MD, USA) with three replicates using the SYBR Green Mix (Thermo, Foster, CA, USA) as previously described [26]. The relative transcriptional level of each tested gene was accurately quantified and calculated according to the 2^–ΔΔCT^ method [27].

### 2.7. Statistical Analysis

All obtained data were statistically analyzed using one-way analysis of variance (ANOVA) using CoStat software with significant differences assessed using least significant difference (LSD) at a *p* ≤ 0.05 level of probability. The significant differences between treatments were plotted, and the standard deviation (±SD) was shown as a column bar. The relative transcriptional levels greater than 1 indicated an increase in gene expression (up-regulation), whereas values less than 1 indicated a decrease in gene expression (down-regulation).

## 3. Results and Discussion

Plant diseases, mainly plant viral infections, are responsible for significant crop losses in agricultural production worldwide, posing severe threats to food security [28]. As biological control agents, plant extracts are considered a sustainable and environmentally friendly alternative to chemical control agents since they contain a diverse set of biologically effective secondary metabolites that might boost systemic resistance of the plant and decrease pathogen development. The present study evaluated the antiviral activity of *P. argentea* methanolic extract against TMV on tomato plants. Moreover, the oxidative and antioxidant enzyme activities and total chlorophyll content were determined in leaves at 21 dpi. Furthermore, the effects of the extract on accumulation levels of TMV-CP and expression levels of five defense-related genes were evaluated.

### 3.1. Effect of P. argentea Extract on TMV Symptoms Development

**Figure 1:**
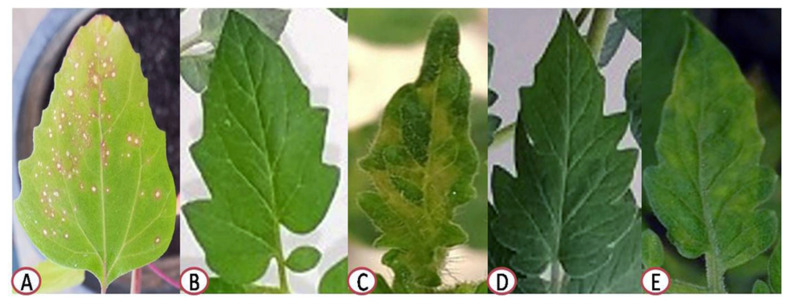
Assay of protective activity of *P. argentea* extract (10 μg/mL) against TMV on *Ch. amaranticolor* at 5 dpi and tomato plants at 21 dpi. (**A**) Development of local lesions on *Ch. amaranticolor* leaf where the half-left side of the leaf was inoculated with TMV without any treatments, while the half-right side was treated with extract; (**B**) mock-treated tomato plants (T1); (**C**) tomato plants inoculated with TMV only (T2); (**D**) tomato plants treated with *P. argentea* extract only (T3); and (**E**) tomato plants treated with *P. argentea* extract 24 h before inoculation with TMV (T4).

**Figure 2:**
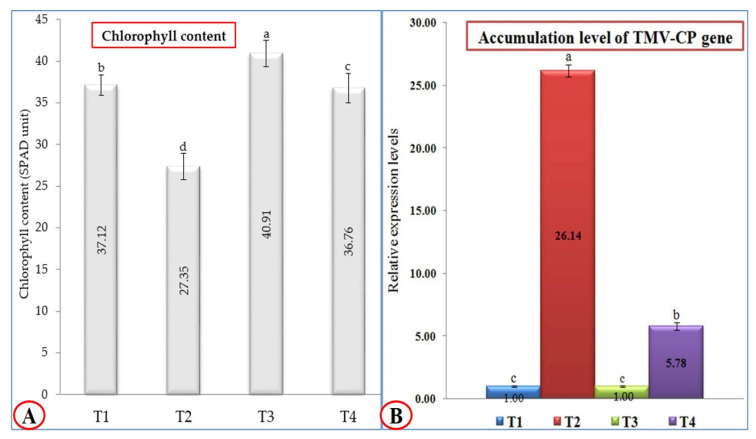
(**A**) Effect of TMV infection and *P. argentea* extract on chlorophyll content (SPAD unit) of tomato plants under greenhouse conditions; (**B**) the relative accumulation level of the TMV-CP gene in different treatments of tomato plants at 21 dpi. T1: Mock-treated tomato plants; T2: tomato plants inoculated with TMV only; T3: tomato plants treated with *P. argentea* extract only, and T4: tomato plants treated with *P. argentea* extract 24 h before TMV inoculation. Columns represent the mean value from three biological replicates, and standard deviation (±SD) is shown in the bars. Columns with the same letter means do not differ significantly.

The extract’s protective activity was primarily checked on *Ch. amaranticolor* plants by counting local lesions that appeared on the infected leaves to investigate its anti-TMV activity. The results showed that the number of local lesions produced by TMV on the right side of leaves, treated with the extract (10 μg/mL) 24 h before viral infection (protective assay), was considerably lower than the number of local lesions that developed on the left side of the leaves that had not been treated (Figure 1A). The protective activity of *P. argentea* extract (10 μg/mL) showed an inhibitory effect of 65.85 ± 2.7% (Figure 1A). Under greenhouse conditions, the foliar application of *P. argentea* extract (10 µg/mL) 24 h before viral inoculation (T4) dramatically reduced disease symptoms (Figure 1), enhanced plant development (Table 2), and decreased accumulation levels of TMV in treated tomato plants (Figure 2) when compared with non-treated plants (T2). At 14 dpi, the TMV-inoculated tomato plants (T2) developed severe mosaic patterns and chlorosis symptoms (Figure 1C) comparable to those previously reported [2,29]. On the other hand, a delay in viral symptoms development for three days in the foliar-treated plants with 10 µg/mL of the extract 24 h before viral infection (T4) was observed (Figure 1E). No symptoms were shown on the mock- (T1) or *P. argentea* extract-treated tomato plants (Figure 1B,D).

### 3.2. Effect of P. argentea Extract on Growth Parameters, Total Chlorophyll Content, and TMV Accumulation Level

The application of *P. argentea* extract, either T3 or T4 treatments, considerably impacted shoot and root system parameters, including length and fresh and dry weights. The statistical analysis of the obtained data revealed that the T3 showed significant increases in all parameters (Table 2). Interestingly, no significant variation was reported between T1 and T4 among growth parameters of shoot systems (Table 2). The TMV infection exhibited substantial decreases in both parameters in untreated tomato plants (T2). The highest shoot length (39.81 cm) was shown in T3, followed by T1, T4, and T2 with 33.41, 26.62, and 33.23 cm, respectively (Table 2). *P. argentea* extract considerably raised the fresh weight of the shoot (10.43 and 7.54 g) and root (5.95 and 4.21 g) systems of T3 and T4 tomato plants, respectively (Table 2). On the other hand, the T2 tomato plants showed significant decreases in fresh weights of the shoot and root (6.72 and 3.12 g) compared to T1 (7.61 and 4.71 g). Similarly, the dry weights of the shoot and root systems of T2 tomato plants were significantly reduced (Table 2). Thus, the increase in tomato plant growth parameters may be related to the ability of *P. argentea* extract to trigger the production of various phytohormones.

Regarding the total chlorophyll content, treatment with *P. argentea* extract only (T3) was the most effective treatment; the tomato plants that underwent this treatment exhibited the highest chlorophyll content (40.91 SPAD unit), followed by mock-treated tomato plants (T1) with 37.12 SPAD unit (Figure 2A). On the other hand, the tomato plants treated with *P. argentea* extract 24 h before inoculation with TMV (T4) showed a significant increase (36.76 SPAD unit) compared to tomato plants inoculated with TMV only (27.35 SPAD unit). Concerning TMV accumulation levels, *P. argentea* extract-treated plants showed a considerable reduction in accumulation levels of TMV (Figure 2B). The qRT-PCR results revealed that the highest accumulation level of TMV-CP (26.14-fold) was reported in T2 plants, while a considerable reduction in viral accumulation level (5.78-fold) in T4 plants was observed (Figure 2B). The significant decrease in TMV accumulation level in T4 tomato leaves by 77.88%, compared to T2 plants, confirmed the protective activity of the *P. argentea* extract against TMV infection. These findings suggest that the *P. argentea* extract may activate the host’s innate immune system and/or induce SAR resulting in TMV inactivation and/or replication inhibition. The results are in agreement with several reports that documented the treatment of tomato and tobacco plants with plant extracts such as *Eupatorium adenophorum*, *Clerodendrum inerme*, *Zingiber officinale*, *Mirabilis jalapa*, and *Mentha longifolia*, associated with SAR induction, TMV particle inactivation, and replication inhibition [30,31,32]. Moreover, the application of *Eucaluptus camaldulensis* on *Nicotiana glutinosa* leaves exhibited inhibitory TMV activity by 72.22% [12].

### 3.3. Oxidative Stress Markers Assay

TMV increased the quick buildup of H_2_O_2_ in infected plants by 2.4 times at 21 dpi (T2, 9.9 µmol/g FW), compared to the non-inoculated control (T1, 4.1 µmol/g FW) (Table 3). In general, the H_2_O_2_ concentration in all treatments was considerably more remarkable than in the control. H_2_O_2_ levels in tomato plants treated with *P. argentea* extract only (T3) and tomato plants treated with *P. argentea* extract 24 h before inoculation with TMV (T4) were 1.26 times compared to the control (5.2 and 5.1 µmol/g FW, respectively). In the T3 and T4 treatments, no significant differences in H_2_O_2_ levels were detected (Table 3). The malondialdehyde (MDA) content (µmol/g FW) was used to determine lipid peroxidation in tomato leaves. When compared to uninoculated controls, virus-inoculated leaves had 2.18 times higher MDA levels (Table 3). In comparison to the control (139 µmol/g FW), MDA buildup was shown to be minimal following T3 and T4 treatments (158 and 161 µmol/g FW), respectively (Table 3). Lipid peroxidation levels in the T3, T4, and T1 treatments were not significantly altered. According to our findings, TMV infection caused a highly significant increase in lipid peroxidation as assessed by MDA. These findings agree with the MDA results obtained by Sobhy et al. [33]. Anthony et al. [34] reported similar findings in bananas infected with *Fusarium oxysporum*. It was reported that the viral infection was linked to an increase in H_2_O_2_ levels [35]. According to Mondal et al. [36], the increased synthesis of peroxidases in infected tomato plant leaves counteracted the higher MDA and H_2_O_2_ levels, preventing tissue oxidation. MDA increase indicates that plants are experiencing high levels of oxidative stress. The MDA could be a great indicator of membrane disruption in plants attacked by pathogens [37]. H_2_O_2_ plays various functional roles in plant-pathogen interactions. It has antimicrobial properties, limits pathogen spread, and functions in the defensin system [38,39,40].

### 3.4. Antioxidant Enzymes Activity

The current investigation discovered a significant rise in three antioxidant enzymes, PPO, CAT, and SOD, in response to tomato inoculation with TMV. The antioxidant enzyme PPO activity of the virus treatment showed the 2-fold highest content with 0.2 µmol/g FW compared with the control (0.1 µmol/g FW). The T3 treatment significantly decreased the enzyme value (by 50% compared to the control). However, there was no significant difference between the enzyme values in T4 and the control treatments (Table 3). PPO has also been found to inhibit the development of plant diseases by producing lignin within the cell wall due to consuming reactive oxygen species (ROS) as a substrate, providing a physical barrier [41]. As shown in Table 3, the virus treatment T2 led to significantly higher levels of accumulated CAT (0.57 µmol/g FW) than the uninoculated control. There was no significant difference between the treatments T1, T3, and T4 in accumulation levels. However, CAT activity values exhibited a slight, insignificant increase in T3 and T4 treatments (0.45 and 0.44 µmol/g FW, respectively) compared to the control treatment (T1, 0.43 µmol/g FW). Following stress exposure, CAT has been shown to play a critical function in preventing oxidative damage and protecting plant cells from oxidative damage caused by ROS [42].

Decreased SOD activity in leaf tissues of all treatments was observed, but this decrease was significantly lower in the infected treatment T2 than in the control treatment T1 (Table 3). On the other hand, the enzyme activity in tomato plants treated with *P. argentea* extract only (T3) and tomato plants treated with *P. argentea* extract 24 h before inoculation with TMV (T4) was 1.18-fold lower than in the un-inoculated control (0.50 and 0.48 µmol/g FW, respectively). Compared to the control, no significant differences in enzyme activity were detected in the T2 and T4 treatments. SOD plays a critical function in limiting pathogen invasion by reinforcing cell walls [43]. According to our results, the PPO, CAT, and SOD activities of tomato plants are considerably affected by *P. argentea* extract treatment before viral inoculation. The high overall phenolic content of *P. argentea* extract and its strong radical quenching capability can be attributed to the results obtained by Sobhy et al. [33]. The antioxidant activities of such different phenolic and flavonoid compounds in *P. argentea* extract might explain why T3 or T4 treatments exhibited low antioxidant enzyme activity. Otherwise, the up-regulation of CAT can minimize oxidative stress produced by H_2_O_2_ and mimic the signaling role of H_2_O_2_ during disease progress [44]. As a result, foliar application of *P. argentea* extract before TMV inoculation might be a potential strategy for minimizing viral infections’ harmful effects. According to our findings, *P. argentea* extract stimulates defense and detoxifying mechanisms, allowing for quicker and more efficient responses to viral inoculation. Furthermore, antioxidant compounds (flavonoids and phenolics) and antioxidant enzyme activity (PPO, CAT, and SOD) may be critical for tomato survival under viral stress. Moreover, the foliar application of *P.* *argentea* extract before TMV inoculation significantly protected tomato plants from viral infection disorders by increasing growth criteria, raising antioxidant status, and regulating gene expression of virus-stressed plants. As a result, this study may have provided ideas for a novel extract–host–pathogen system, albeit the specific mechanism of this process is yet unknown. Understanding the proteome changes in extract-primed tomato will require more investigations later.

### 3.5. Transcriptional Levels of Defense-Related Genes

#### 3.5.1. Pathogenesis-Related Proteins

**Figure 3:**
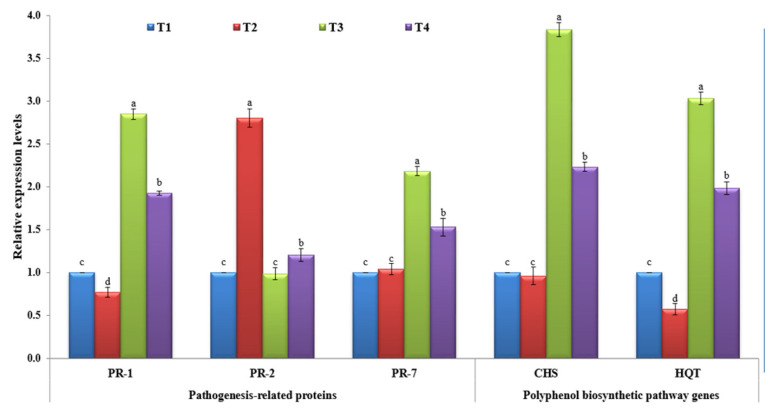
The relative expression levels of five tested tomato genes (PR-1, PR-2, PR-7, CHS, and HQT) at 21 dpi of different treatments. T1: mock-treated tomato plants; T2: tomato plants inoculated with TMV only; T3: tomato plants treated with *P. argentea* extract only, and T4: tomato plants treated with *P. argentea* extract 24 h before TMV inoculation. Columns represent the mean value from three biological replicates, and standard deviation (±SD) is shown in the bars. Columns with the same letter means do not differ significantly.

Many reports suggest that the collective set of pathogen-related (PR) proteins are responsible for SAR as well as being effective at preventing pathogen development, multiplication, and/or spread [45,46]. In the current study, the three PR genes (PR-1, PR-2, and PR-7) were evaluated upon TMV infection at different treatments. For PR-1, it was shown that the non-treated tomato plants challenged with TMV only (T2) showed a significant (*p* ≤ 0.05) decrease in PR-1 level, with a 0.77-fold lower change in relative expression level than the control (T1) (Figure 3). On the other hand, the tomato plants treated with *P. argentea* extract alone (T3) or *P. argentea* extract +TMV (T4) exhibited significant up-regulation of PR-1 with relative expression levels 2.85- and 1.92-fold, respectively, higher than the control (Figure 3). It is well known that salicylic acid (SA) is a key plant signal phytohormone molecule, and its involvement in plant immunity activation has been reported for more than two decades [47,48]. Meanwhile, after pathogen infection, induction of PR-1, a SA marker gene, is frequently associated with SA activation [48,49]. Consequently, we suggest that the *P. argentea* extract contains elicitor metabolite compounds capable of activating SAR and increasing plant resistance against viral infection. Besides its main role in the cell-to-cell spread of virus movement, PR-2 encoding β-1,3-glucanases mediates cell-to-cell communication and long-distance signaling by restricting callose deposition near plasmodesmata (PD) [50,51,52]. It was shown that TMV elevates the activity of PR-2 in order to facilitate its movement through plant cells [17,53]. The obtained results are in agreement with former studies that have shown a considerable activation of PR-2 upon viral infections of different plant species, including *Arabidopsis*, tobacco, potato, and tomato [17,54,55,56,57]. Furthermore, a deficiency of tobacco PR-2 reduces susceptibility to viral infection [57], whereas overexpression speeds up the spread of PVY infection across cells [58,59]. Interestingly, the treatments of tomato plants with *P. argentea* extract before viral infection (T4) exhibited slight increases in PR-2, a 1.20-fold higher change than the control (Figure 3). There were no significant changes reported between T3 tomato plants and the controls (*p* ≤ 0.05). Thus, the foliar application of *P. argentea* extract may decrease TMV infection by reducing PR-2 expression and preventing long-distance viral movement between cells. PR-7 is the most conspicuous PRs gene in the tomato plant tissues that encodes plant endoproteinase activity. It is an important component of defense response proteins and is implicated in microbial cell wall disintegration [45,60,61]. In this study, it was reported that the application of *P. argentea* extract either alone (T3) or before viral infection (T4) was associated with the induction of PR-7 at 21 dpi (Figure 3). The T3 tomato plant exhibited the highest expression level (2.18-fold), while T4 showed a 1.53-fold greater change in relative expression level than the control (Figure 3). On the other hand, no significant change was reported in the T2 tomato plant compared to the control at *p* ≤ 0.05 (Figure 3). These results agree with previous studies that have reported up-regulation of tomato PR-7 upon plant pathogen infections, including TMV and AMV infestation [17,61,62]. However, more characterization and functional analysis of PR-7 will lead to a better understanding of TMV–tomato interactions.

#### 3.5.2. Polyphenolic Biosynthetic Pathway

Polyphenolic compounds are among the secondary metabolites crucial for plant growth, development, and tolerance to biotic and abiotic stresses, including viruses [63]. Upon plant infection, they are transported to the infection sites, trigger hypersensitive responses, and induce programmed cell death [64,65]. Chalcone synthase (CHS) is the first enzyme in the flavonoid metabolic pathway responsible for transforming *p*-coumaroyl CoA to naringenin chalcones, a strict precursor for flavonoids in various plant tissues [66,67]. Compared to the control (T1), the challenged tomato plants either in tomato plants treated with *P. argentea* extract only (T3) or tomato plants treated with *P. argentea* extract 24 h before inoculation with TMV (T4) exhibited a considerable accumulation level of CHS inside the plant tissues (Figure 3). The T3 tomato plant showed the highest transcription level (3.84-fold), followed by T4 plants with a relative expression level 2.23-fold higher than that of the control (Figure 3). On the other hand, the tomato plants inoculated with TMV only (T2) exhibited slightly decreased relative expression of CHS with no significant changes compared to the control (*p* ≤ 0.05). Chlorogenic acid (CGA) is one of the most valuable polyphenolic compounds that plays a crucial role in improving plant resistance and suppressing pathogens such as viruses [68,69]. HQT is a principal enzyme in CGA biosynthesis, where it catalyzes the transformation of caffeoyl-CoA to quinic acid [70]. In the current study, the HQT was suppressed and down-regulated in tomato plant tissues challenged with TMV only (T2) with a relative expression level 0.57-fold lower than that of the control (Figure 3). In this context, TMV was reported to suppress CGA biosynthesis inside infected tomato tissues [71]. Intriguingly, the application of *P. argentea* extract induced HQT transcripts in both T3 and T4 treatments. A higher transcriptional level of HQT (3.03-fold) was shown in T3 tissues, while the T4 tomato plant exhibited a 1.98-fold higher change in relative expression level than the control (Figure 3). It was reported that the overexpression of HQT was correlated with the accumulation of chlorogenic acid content and vice versa inside plant tissues [28,70]. In line with the obtained results, the foliar application of *P. argentea* extract induced the transcriptional of both CHS and HQT, resulting in polyphenolic compound accumulation inside treated plant tissues, developing SAR, and increasing resistance against TMV infection.

### 3.6. HPLC Analysis of P. argentea Extract

**Figure 4:**
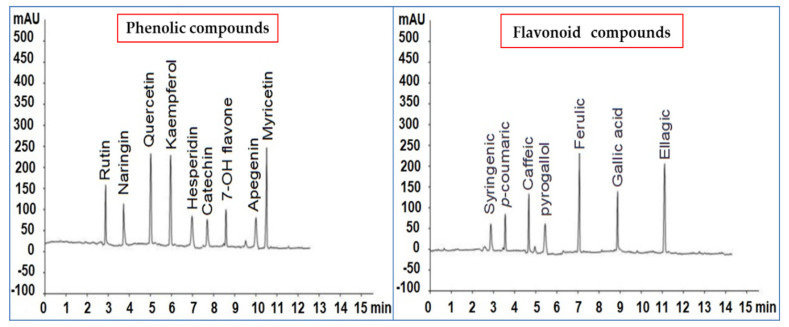
Phenolic and flavonoid compound chromatograms were identified in *Paronychia argentea* methanolic extract.

The HPLC fingerprint of phytochemical compounds from the aerial parts of *P. argentea* (genus *Paronychia*, family *Caryophyllaceae*) is presented in Figure 4. The phenolic compounds identified in µg/mL were syringenic acid (2.33), *p*-coumaric acid (3.05), caffeic acid (4.55), pyrogallol acid (1.45), ferulic acid (8.69), gallic acid (5.36), and ellagic acid (7.89). The flavonoid compounds in µg/mL were rutin (5.66), naringin (4.12), quercetin (7.44), kaempferol (7.39), hesperidin (2.55), catechin (2.46), 7-OH flavone (3.56), apigenin (2.13), and myricetin (8.36). The primary–secondary metabolites of Caryophyllaceae are phytoecdysteroids, saponins, other sterols, lignans, flavonoids, other polyphenols, essential oils, vitamins, alkaloids, and cyclic peptides. *Paronychia chionaea* synthesized the triterpene saponins constituting gypsogenin, gypsogenic acid, and quillaic acid [72]. In comparison, *Paronychia virginica* contains the sterol-type class of compounds, D7-sterols, represented by 22-dihydrospinasterol [73]. Meanwhile, Barca et al. [74] identified six flavones in the methanol extract of the aerial parts of *P. argentea* by NMR and ESI-MS; isorhamnetin 3-O-b-D-glucoside, nepetin, quercetin 3-O-((2,6-acetyl)-b-D-glucosyl)-(1-6)-b-D-galactoside, quercetin 3-O-b-D-galactoside (hyperoside), quercetin 3-O-b-D-glucosyl-(1-6)-b-D-galactoside 7-(b-D-glucosyl)-40, 5-dihydroxy-30, and 6-dimethoxy flavone (jaceoside). The same compounds were identified by Sait et al. [75] in the ethanolic extract of Algerian *P. argentea* aerial parts analyzed by HPLC-UV/DAD and ESI-MS(n). The identified compounds were quercetin-3-O-glucoside, isorhamnetin-3-O-dihexoside, quercetin methyl ether-O-hexoside, quercetin, isorhamnetin, and jaceosidin. In the same way, our methanolic *P. argentea* aerial parts extract had a higher content of the flavonoid compound quercetin. Meanwhile, the aqueous extract of Syrian *P. argentea* showed the highest phenol content, while the antioxidant ability was higher in the methanolic extract.

The high concentration of phenolic acids and flavonoids in *P. argentea* extract may have reduced stress markers detected in this study. According to Arora et al. [76], flavonoids can change peroxidation kinetics by altering the lipid pecking order. Flavonoids help keep membranes stable by reducing membrane fluidity, preventing free radical dispersal, and minimizing peroxidation of plant cell walls [77]. The accumulation of flavonoids, which are antioxidant compounds, was increased in response to TMV inoculation. Following an infection, flavonoids scavenge ROS generated by pathogens and the plant, forming a physical barrier against pathogen attack [78]. By incorporating them into the cellulose component of the cell walls, phenolics may enhance plant resistance to pathogen invasion [79]. The findings of our investigation revealed that treating tomato plants with *P. argentea* extract delayed and decreased the adverse effects of TMV infection and alleviated the oxidative stress produced by viral inoculation. The presence of phenolic or flavonoid compounds such as caffeic acid, *p*-coumaric acid, ferulic acid, ellagic acid, rutin, kaempferol, naringenin, quercetin, and myricetin in the extract might explain our findings, as reported by several authors [16,80,81].

The most abundant phenolic acid found in *P. argentea* extract was ferulic acid (FA), a common natural phytochemical found in leaves and seeds free and covalently conjugated to glycoproteins, polysaccharides, polyamines, hydroxy fatty acids, and lignin. FA is required for the stiffness of the cell wall and acts as an antioxidant, antimicrobial, anticarcinogenic, antiviral, and enzyme activity modulator [82]. The application of ferulic acid amide derivatives at 500 μg/mL exhibited higher protective and curative activities against TMV than the ribavirin (control) compound [83,84]. Caffeic acid is a hydroxycinnamic acid with many acrylic functional groups and is most commonly produced from plants. Caffeic acid improves plant stress tolerance primarily due to its high antioxidant activity and antioxidant enzyme modulation and helps to scavenge reactive oxygen species. Caffeic and its derivatives play a role in dealing with salinity, ion toxicity, drought, heavy metal stress, and treating pathogen attacks caused by bacteria, fungi, and viruses [85,86]. According to Davidson [87], exogenous caffeic acid application reduced the development of several *Fusarium* and *Saccharomyces* species when 500 μg/mL was applied. Many polyphenols such as caffeic acid, ellagic acid, catechins, chlorogenic acid, gallic acid, quercetin, ferulic acid, and myricetin have antibacterial, antiviral, anti-inflammatory, anti-cancer, and antioxidant properties [88,89]. We suggest that such identified polyphenolic compounds could work as elicitor molecules and play significant roles in SAR.

Generally, plant and herbal extracts may differ according to the type of extraction solvent, extraction period, and temperature used. Moreover, the phytochemical constituents of the same plant/herbal extract or a product are likely to differ greatly between batch-es and manufacturers [90]. Herbs contain a variety of compounds, many of which have yet to be identified, and many of which lack an identification component. This makes it difficult to standardize botanicals, while some can be manufactured to include a consistent amount of a crucial component or class of components, such as ginsenosides in ginseng products or anthocyanins in bilberry products. Even if important substances have been discovered and a standard content has been agreed upon or suggested, there is no guarantee that particular commercial products will contain them. Consequently, many monographs and guidelines on good collection practices for herbal origin starting materials as well as guidelines on the standardization of applications and setting up pragmatic approaches for identifying and quantifying herbal preparations and their complex compositions have all been produced [91,92]. Furthermore, future steps of research will also have to focus on the standardization of the extract, making sure that the effects are reproducible when starting from different materials.

## 4. Conclusions

Based on the outcome of our investigation, it is possible to conclude that the foliar application of a methanolic extract of *P. argentea* (10 µg/mL) enhanced tomato plant growth, induced systemic resistance, reduced TMV accumulation level, and increased total chlorophyll contents. Compared to non-treated plants, significant reductions (77.88%) in TMV accumulation levels with elevations in the production of PPO, CAT, and SOD and transcriptional levels of PR-1, PR-7, CHS, and HQT in *P. argentea*-treated plants were reported. HPLC analysis revealed that the polyphenolic compounds of *P. argentea* extract with the highest contents were gallic acid, kaempferol, quercetin, ellagic acid, myricetin, and ferulic acid. The obtained results indicated that *P. argentea* extract had an inhibitory effect against TMV infection. However, further examinations of the antiviral activities of polyphenolic compounds are needed for open-field applications and commercial uses.

## Figures and Tables

**Table 1:** Target genes and primer sequences used in the quantitative real-time PCR analysis.

Target Gene	Primer Code	Direction	Nucleotide Sequences (5’-3′)
Tobacco mosaic virus-coat protein	TMV-CP	Sense	ACGACTGCCGAAACGTTAGA
		Antisense	CAAGTTGCAGGACCAGAGGT
Pathogenesis related protein-1	PR-1	Sense	CCAAGACTATCTTGCGGTTC
		Antisense	GAACCTAAGCCACGATACCA
*β*-1,3-glucanases	PR-2	Sense	TATAGCCGTTGGAAACGAAG
		Antisense	CAACTTGCCATCACATTCTG
Proteinase	PR-7	Sense	AACTGCAGAACAAGTGAAGG
		Antisense	AACGTGATTGTAGCAACAGG
Chalcone synthase	CHS	Sense	CACCGTGGAGGAGTATCGTAAGGC
		Antisense	TGATCAACACAGTTGGAAGGCG
Hydroxycinnamoyl Co A: quinate hydroxycinnamoyl transferase	HQT	Sense	CCCAATGGCTGGAAGATTAGCTA
		Antisense	CATGAATCACTTTCAGCCTCAACAA
Beta-actin	*β*-actin	Sense	ATGCCATTCTCCGTCTTGACTTG
		Antisense	GAGTTGTATGTAGTCTCGTGGATT

**Table 2:** Effect of different treatments with *P. argentea* extract on growth parameters of tomato plants under greenhouse conditions.

Treatment	Shoot			Root		
	Length (cm)	Fresh Weight (g)	Dry Weight (g)	Length (cm)	Fresh Weight (g)	Dry Weight (g)
T1	33.41± 2.39 b	7.61± 1.97 b	3.13± 0.73 b	15.91 ± 0.74 b	4.71 ± 0.89 b	2.13 ± 0.50 b
T2	26.62 ± 1.30 c	6.72 ± 2.48 c	2.71 ± 0.57 c	10.52 ± 0.79 d	3.12 ± 0.40 d	1.74 ± 0.36 d
T3	39.81 ± 2.34 a	10.43 ± 1.98 a	3.52 ± 0.38 a	18.43 ± 3.20 a	5.95 ± 1.29 a	2.63 ± 0.49 a
T4	33.23 ± 1.98 b	7.54 ± 1.91 b	3.05 ± 0.25 b	13.42 ± 1.68 c	4.21 ± 1.14 c	2.01 ± 0.28 b

T1: Mock-treated tomato plants; T2: tomato plants inoculated with TMV only; T3: tomato plants treated with *P. argentea* extract only and T4: tomato plants treated with *P. argentea* extract 24 h before TMV inoculation. Data represent the mean value from three biological replicates, and standard deviation (±SD) is shown. Data with the same letter means do not differ significantly. The significant differences were assessed using the least significant difference (LSD) at a *p* ≤ 0.05 level of probability.

**Table 3:** Effect of different treatments on the activity of the enzymatic markers of tomato plants under greenhouse conditions.

Treatments	Oxidative Stress Markers		Antioxidant Enzymes Activity		
	MDA	H_2_O_2_	PPO	CAT	SOD
	µmol/g FW		µmol/g FW		
T1	139 ± 21 b	4.1 ± 0.75 c	0.1 ± 0.01 b	0.43± 0.01 b	0.59 ± 0.01 a
T2	304 ± 33 a	9.9 ± 0.43 a	0.2 ± 0.01 a	0.57± 0.02 a	0.41 ± 0.01 c
T3	158 ± 6.4 b	5.2 ± 0.47 b	0.05 ± 0.01 c	0.45 ± 0.02 b	0.50 ± 0.08 b
T4	161 ± 31 b	5.1 ± 0.30 b	0.09 ± 0.01 b	0.44 ± 0.02 b	0.48 ± 0.03 bc

T1: Mock-treated tomato plants; T2: tomato plants inoculated with TMV only; T3: tomato plants treated with *P. argentea* extract only, and T4: tomato plants treated with *P. argentea* extract 24 h before TMV inoculation. Data represent the mean value from three biological replicates, and standard deviation (±SD) is shown. Data with the same letter means do not differ significantly. The significant differences were assessed using the least significant difference (LSD) at a *p* ≤ 0.05 level of probability.

## Data Availability

All data reported here are available from the authors upon request.
